# Supplementary material for: Ionomers Based on Addition and Ring Opening Metathesis Polymerized 5-phenyl-2-norbornene as a Membrane Material for Ionic Actuators
Source: Membranes (Basel). 2022 Mar 10;12(3):316. doi: 10.3390/membranes12030316 (PMC8953079; doi:10.3390/membranes12030316)
Supplement: Supplementary file 1 [file membranes-12-00316-s001.zip › membranes-1597511 - SM.pdf]

Supplementary information for

Ionomers based on addition and ring opening metathesis polymerized 5-phenyl-2-norbornene as a membrane material for ionic actuators

Oleg S. Morozov[1*], Alexander V. Babkin[1], Anna V. Ivanchenko[1], Svetlana S. Shachneva[2], Sergey S. Nechausov[1], Dmitry A. Alentiev[3], Maxim V. Bermeshev[3], Boris B. Bulgakov[1] and Alexey V. Kepman[1]

1 Department of Chemistry, Lomonosov Moscow State University, Moscow 119991, Russia
2 Faculty of Materials Science, Lomonosov Moscow State University, Moscow 119991, Russia
* Correspondence should be addressed to Oleg S. Morozov; osmorozov@yandex.ru

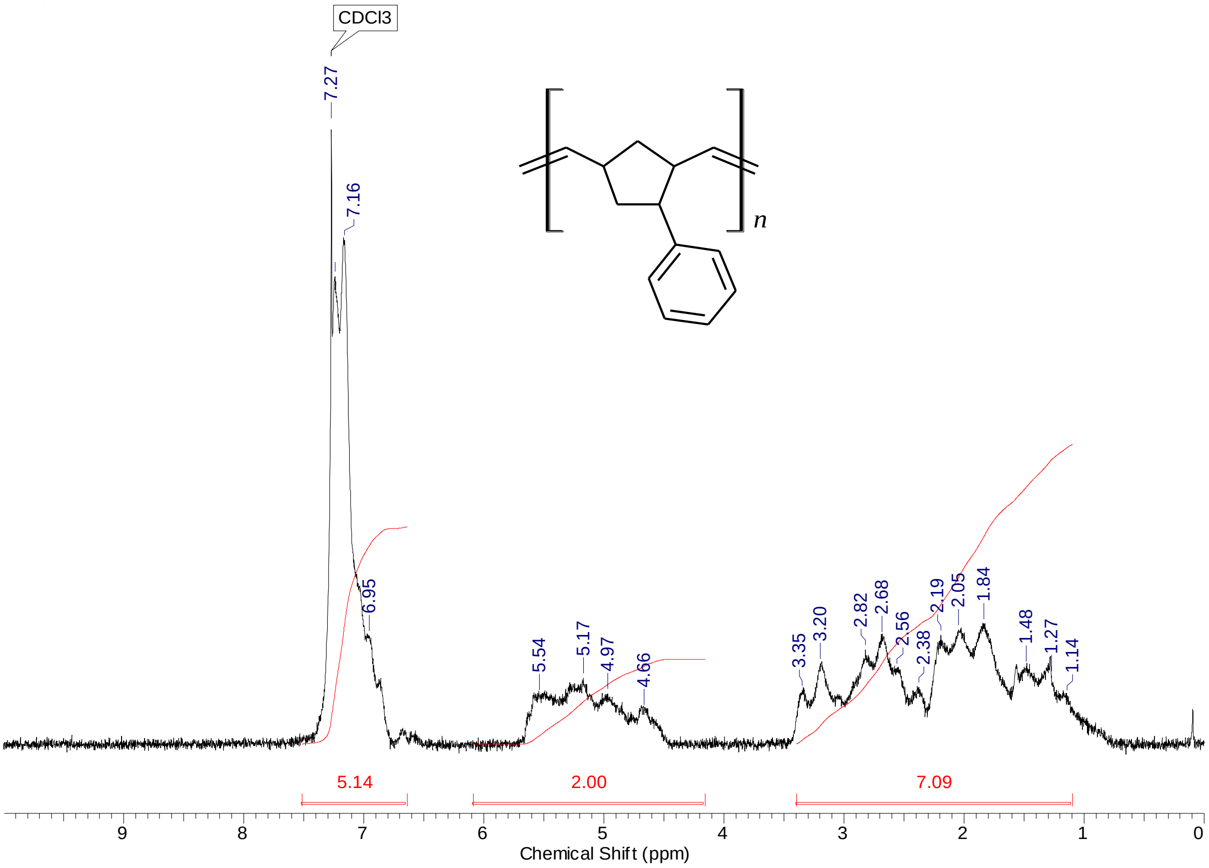

FigureS1. 1H NMR spectrum of poly(phenyl norbornene) (PPhNB)

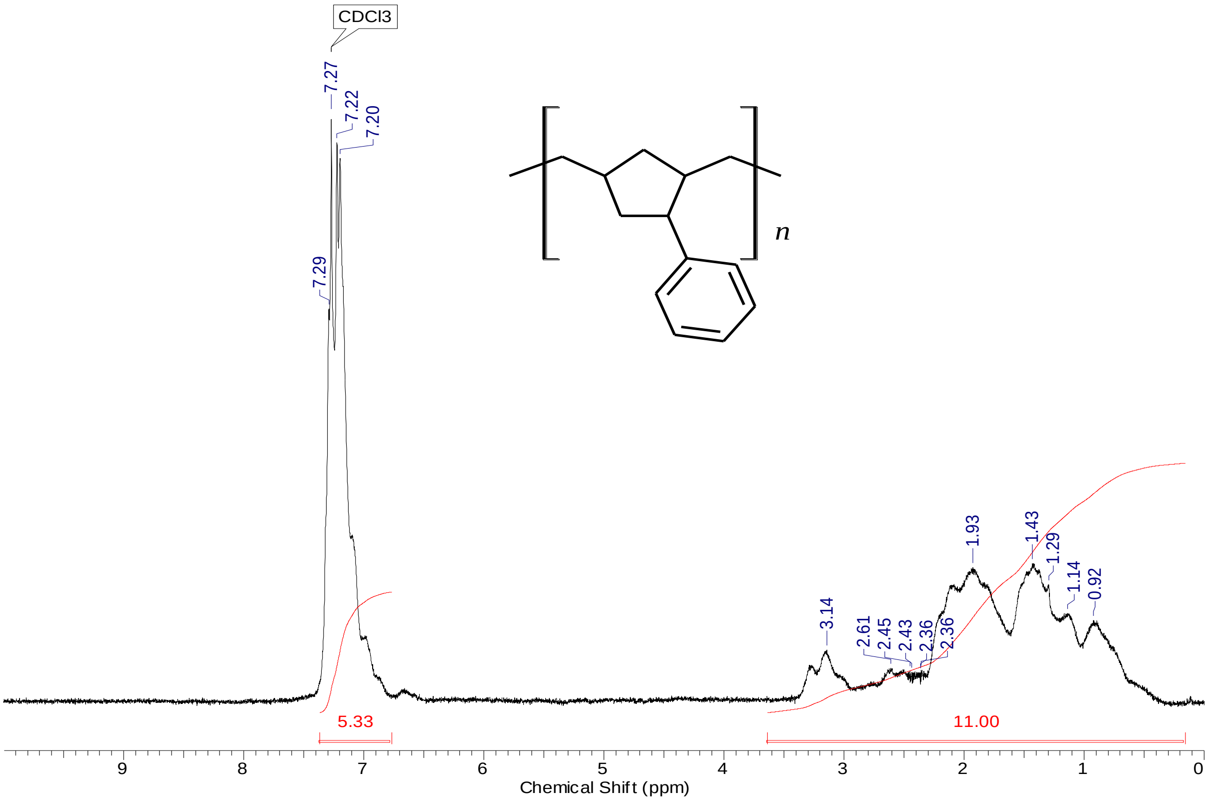

FigureS2. 1H NMR spectra of hydrogenated poly(phenyl norbornene) (HPPhNB)

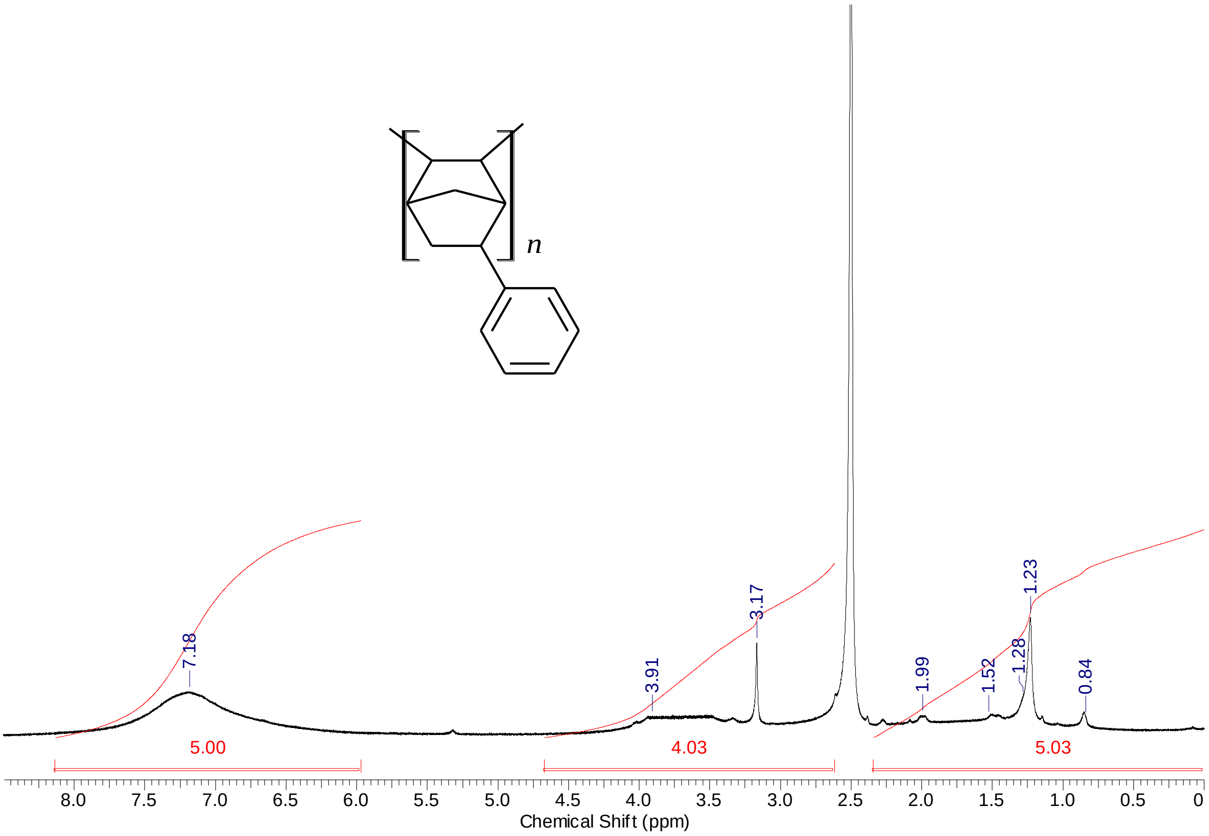

FigureS3. 1H NMR spectra of poly(phenyl norbornene) (APPhNB)

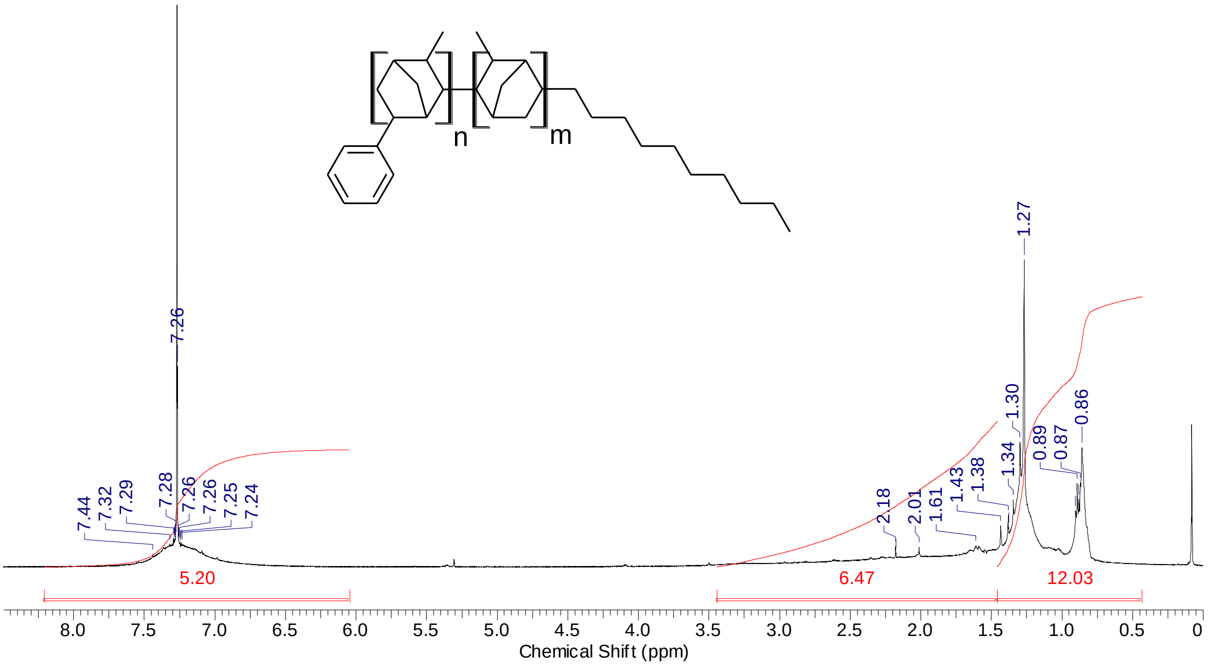

FigureS4. 1H NMR spectra of block-copolymer

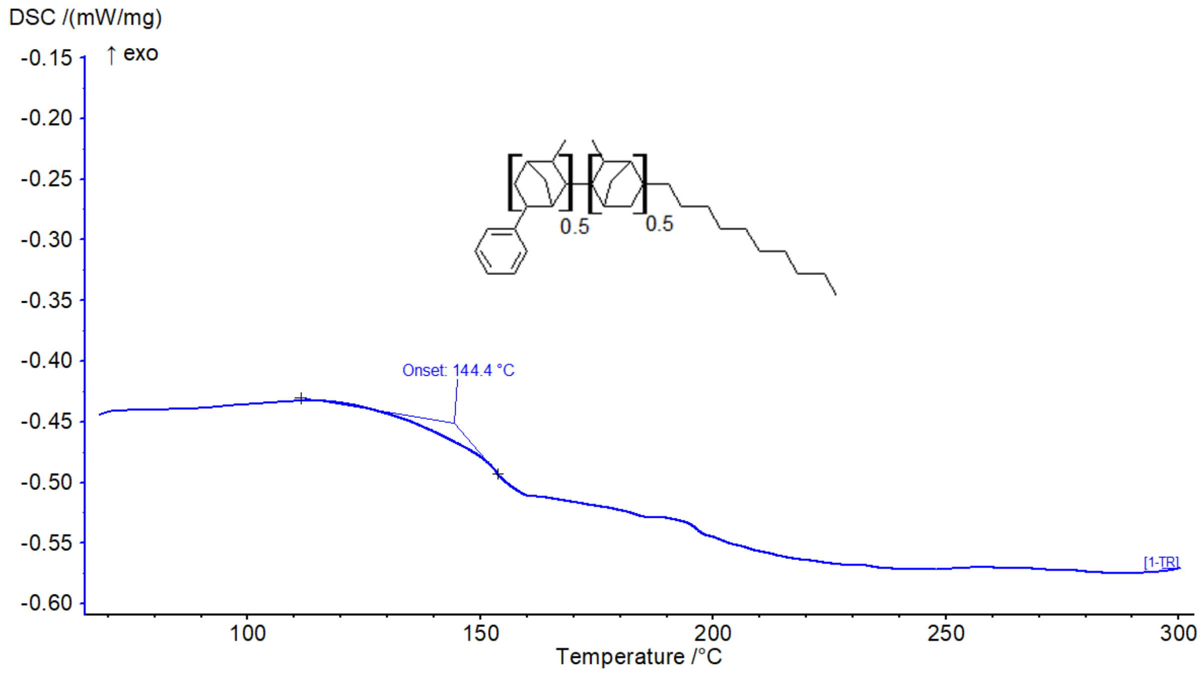

FigureS5. DSC curve of block-copolymer

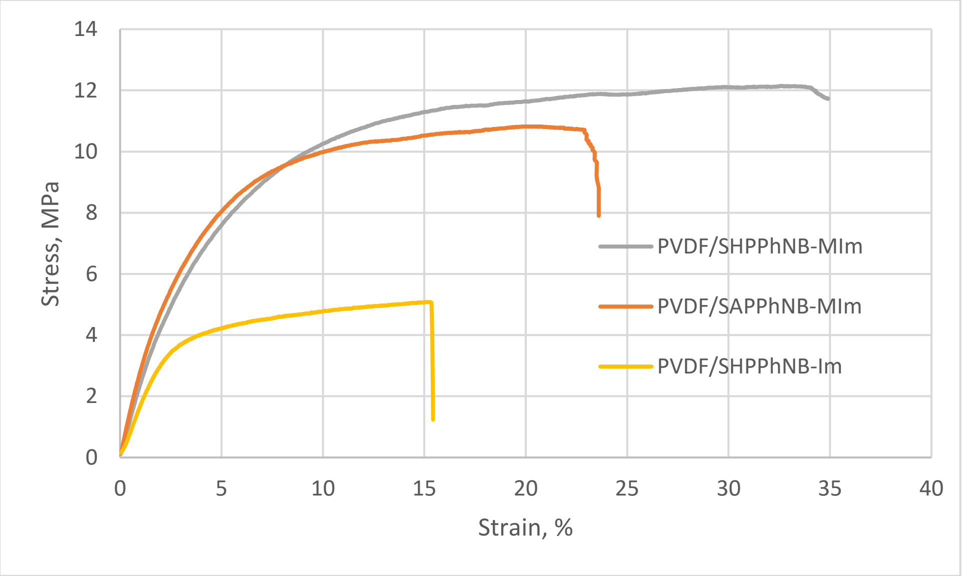

Figure S6. Stress–strain curves of PVDF blend membranes

Table S1. Properties of used SWCN based electrodes

Electrolyte	Modulus, MPa	Tensile strength, MPa	Fracture strain, %	Thickness, µm
Dry	757	27.4	14	25
Swollen in IL	490	17.7	14	25

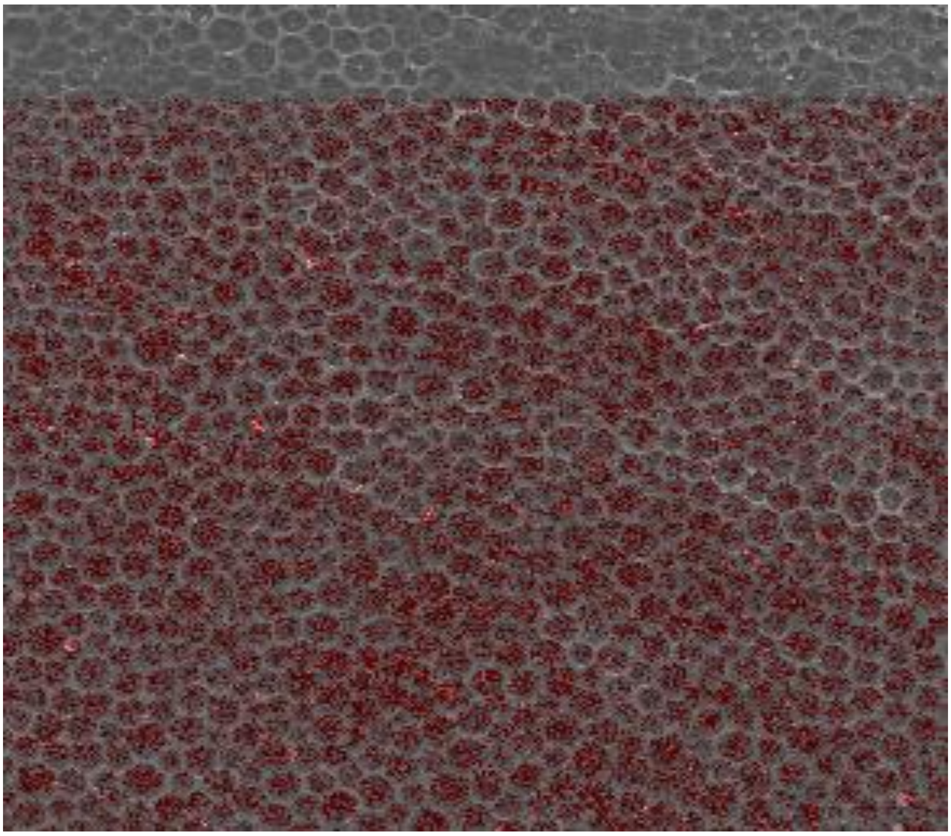

Figure S7. EDX sulfur mapping of PVDF/SHPPhNB-Im membrane

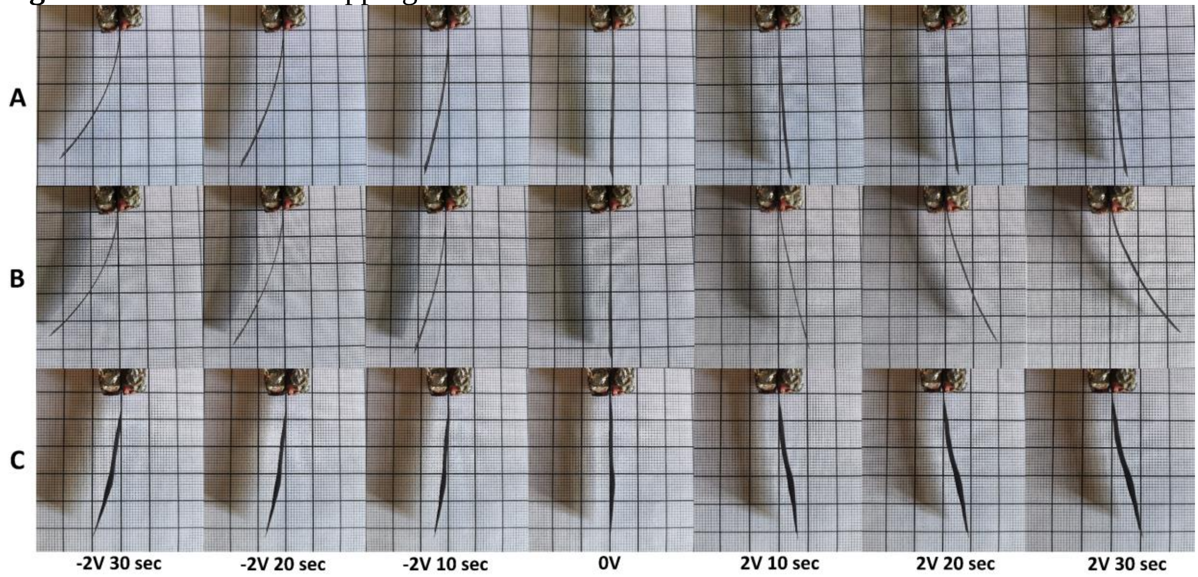

Figure S8. Photographs of bending of actuators based on PVDF/SHPPhNB-Im (A), PVDF/SHPPhNB-MIm (B) and classical Bucky gel actuator (C)

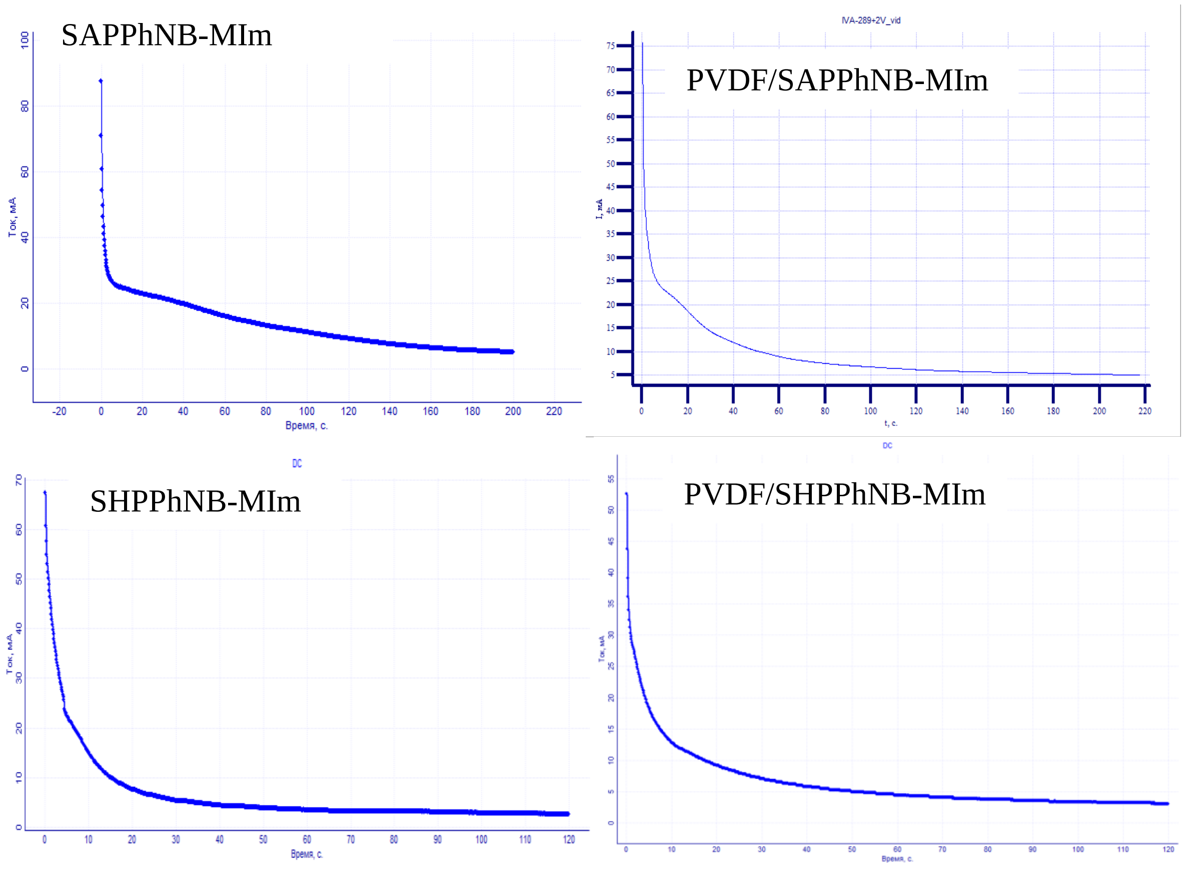

Figure S9. Time dependence of current of PVDF/ SAPPhNB -MIm, PVDF/SHPPhNB-MIm and SHPPhNB-MIm under 2V DC

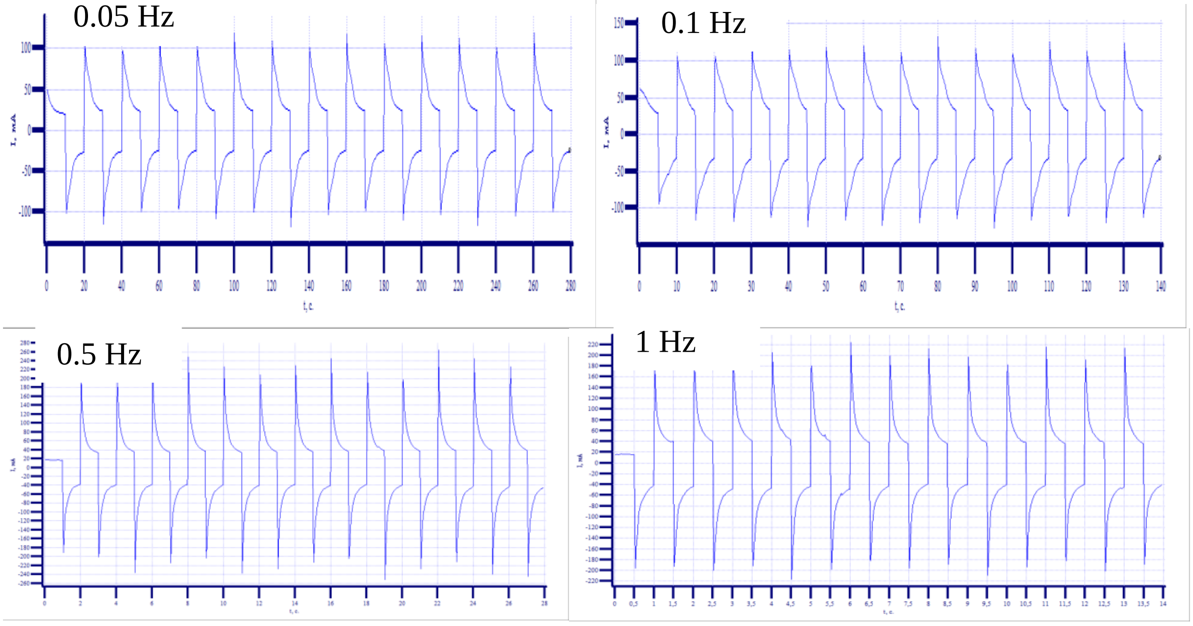

Figure S10. Time dependence of current of SAPPhNB-MIm under2 V at 0.05, 0.1, 0.5, 1 Hz

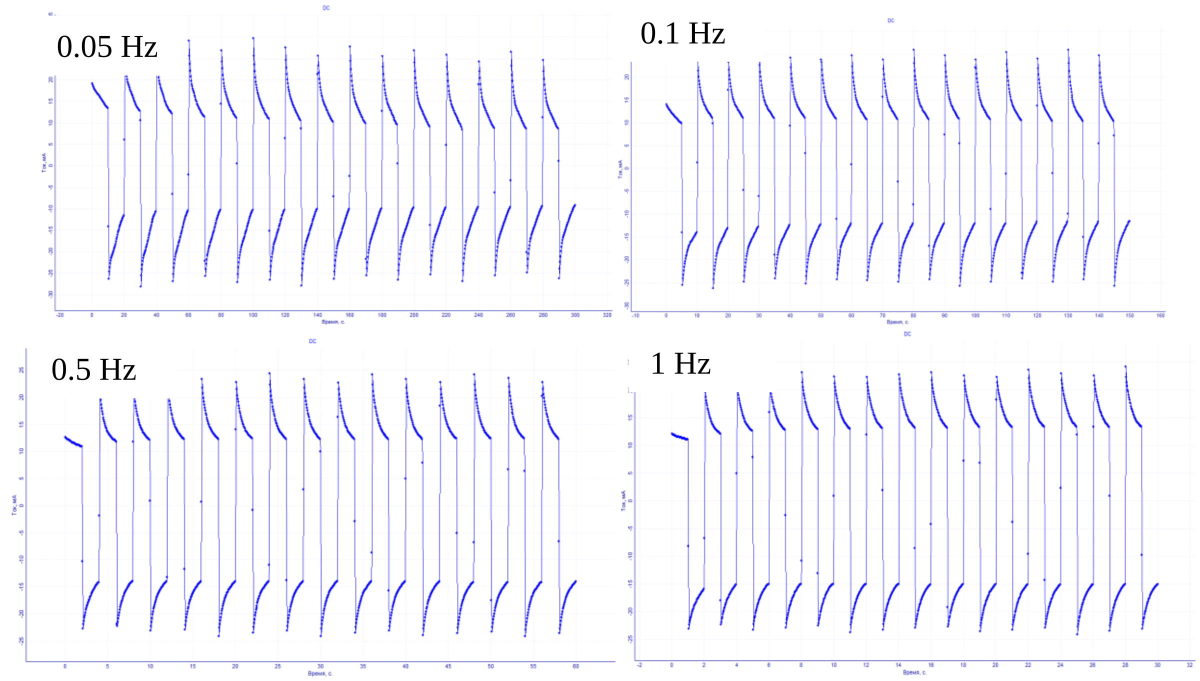

Figure S11. Time dependence of current of PVDF/SAPPhNB -MIm under 2 V at 0.05, 0.1, 0.5, 1 Hz

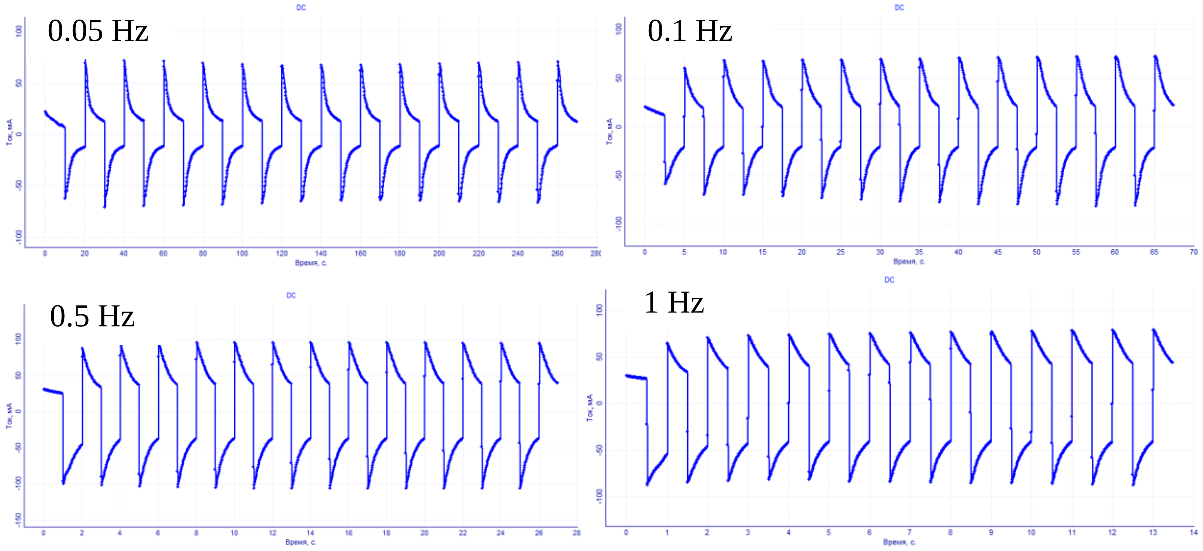

Figure S12. Time dependence of current of PVDF/SHPPhNB-MIm under 2 V at 0.05, 0.1, 0.5, 1 Hz

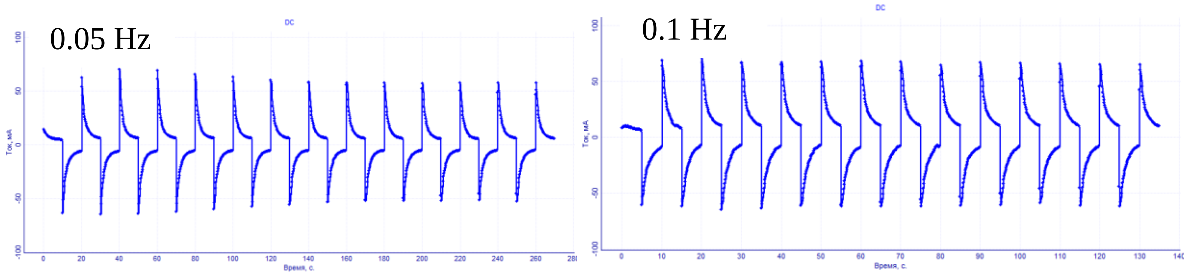

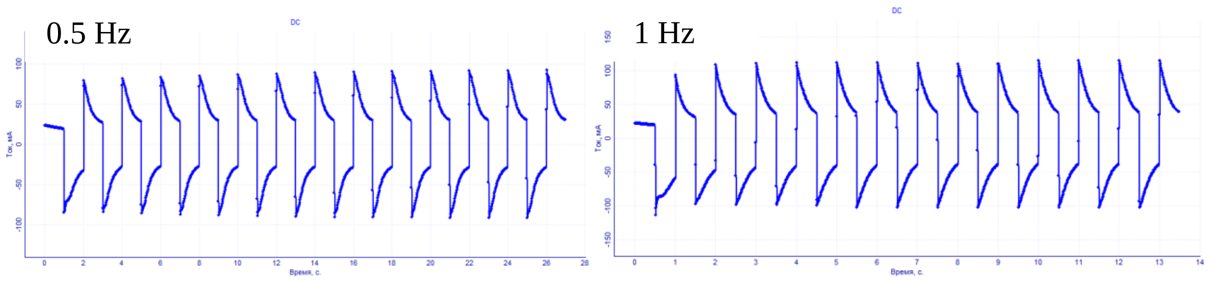

Figure S13. Time dependence of current of SHPPhNB-MIm under 2 V at 0.05, 0.1, 0.5, 1 Hz

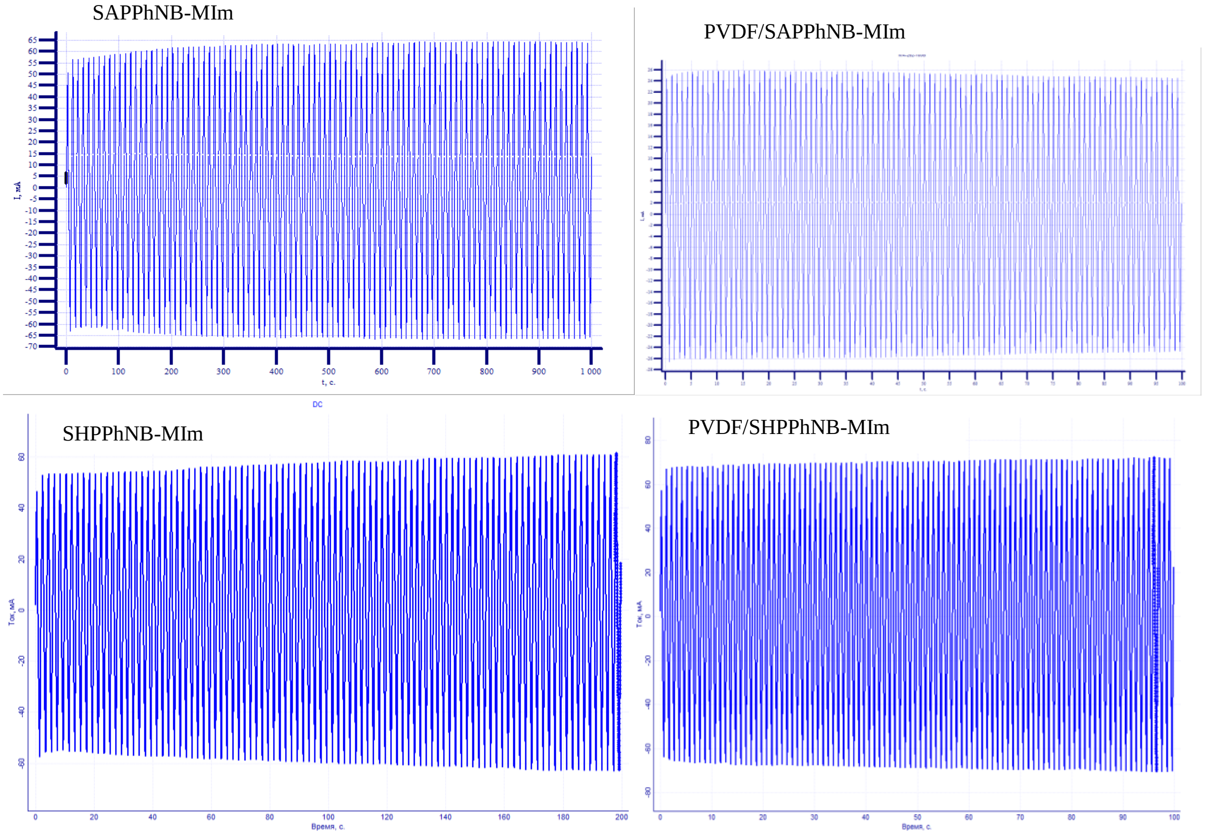

Figure S14. Time dependence of current of the actuators during 100 cycles at 0.5 Hz

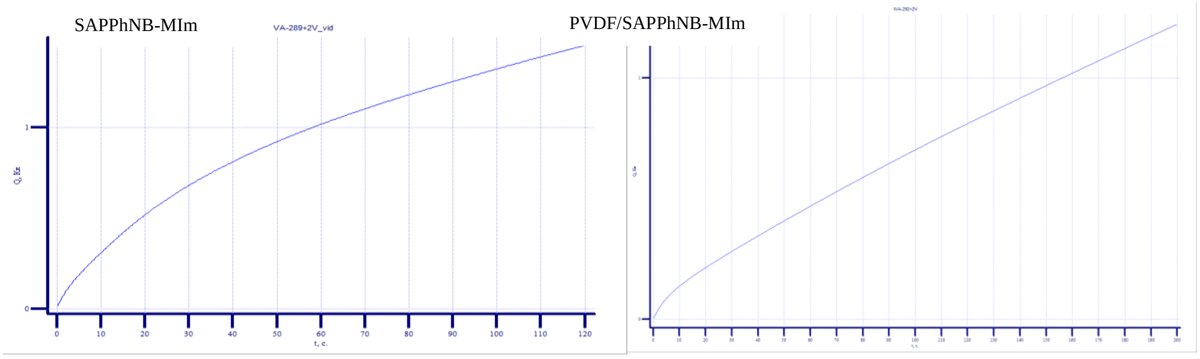

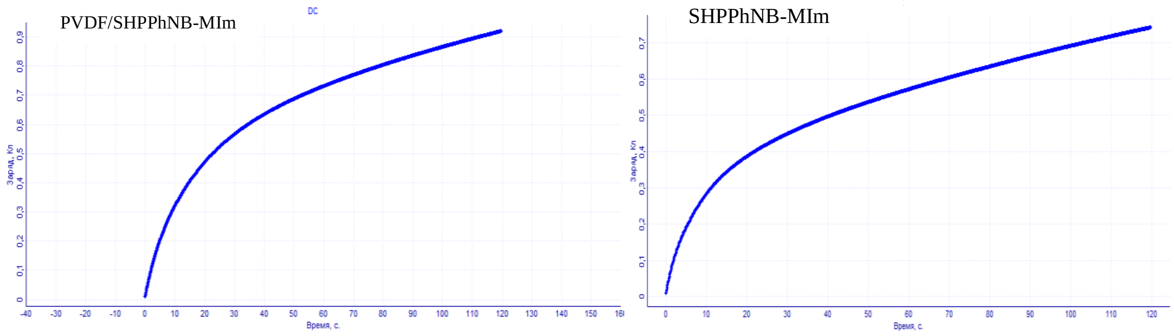

Figure S15. Time dependence of charge of the actuators under 2 V DC

Details on strain and blocking force measurements

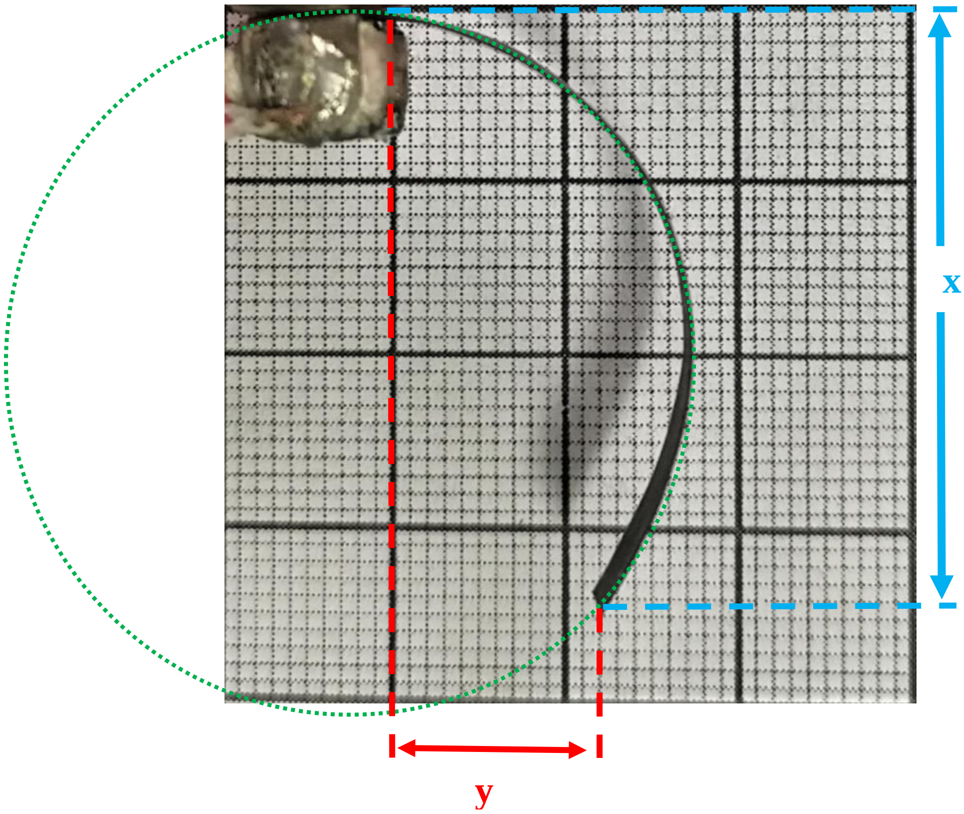

To measure bending, the actuator was placed over graph paper and when voltage was turned on recording on the digital camera started. The deformation of the actuators was calculated under the assumption that the shape of the sample during bending is an arc of a circle. The strain was calculated using the following formula: $\varepsilon(\%) = \frac{2hx}{x^2+y^2}100\%$, x, y are the coordinates of the tip of the actuator, h is actuator thickness. The thickness of the actuator was measured in several places with a micrometer; the smallest value was used for calculations. For measurements at high frequency, recording was carried out at a speed of 240 frames per second. The peak-to-peak strain was calculated for the extreme positions independently and the result was summed.

The generated blocking force was measured using analytical balance. A load with a hook was placed on the balance and the actuator was fixed horizontally, so that its tip touched the hook. After switching on the voltage, the weight values of the scales were recorded by digital camera and the value of the blocking force was calculated by the formula: F=-mg. All actuators were installed so that the distance from the connection point of the actuator to the load was 40 mm.

The polarity was set so that the force acted against the weight. Otherwise, the actuators lost their rigidity due to Joule heating and the weight of the actuator would be added to the generated force.
